# Temperature Effects of Nuclear and Electronic Stopping Power on Si and C Radiation Damage in 3C-SiC

**DOI:** 10.3390/ma17122843

**Published:** 2024-06-11

**Authors:** Ewelina Kucal, Przemysław Jóźwik, Cyprian Mieszczyński, René Heller, Shavkat Akhmadaliev, Christian Dufour, Konrad Czerski

**Affiliations:** 1 National Centre for Nuclear Research, A. Soltana 7, 05-400 Otwock-Swierk, Poland; przemyslaw.jozwik@ncbj.gov.pl (P.J.); cyprian.mieszczynski@ncbj.gov.pl (C.M.); konrad.czerski@usz.edu.pl (K.C.); 2 Helmholtz-Zentrum Dresden-Rossendorf, Bautzner Landstr. 400, 01328 Dresden, Germany; r.heller@hzdr.de (R.H.); akhmadal@hzdr.de (S.A.); 3 Centre de Recherche sur les Ions, les Matériaux et la Photonique, 6 Bvd du Maréchal Juin, CEDEX 4, 14050 Caen, France; christian.dufour@ensicaen.fr; 4 Institut für Festkörper-Kernphysik gGmbH, Leistikowstraße 2, 14050 Berlin, Germany; 5 Institute of Physics, University of Szczecin, ul. Wielkopolska 15, 70-451 Szczecin, Poland

**Keywords:** radiation damage, stopping power, Rutherford backscattering, ion channeling

## Abstract

Silicon carbide has been considered a material for use in the construction of advanced high-temperature nuclear reactors. However, one of the most important design issues for future reactors is the development of structural defects in SiC under a strong irradiation field at high temperatures. To understand how high temperatures affect radiation damage, SiC single crystals were irradiated at room temperature and after being heated to 800 °C with carbon and silicon ions of energies ranging between 0.5 and 21 MeV. The number of displaced atoms and the disorder parameters have been estimated by using the channeling Rutherford backscattering spectrometry. The experimentally determined depth profiles of induced defects at room temperature agree very well with theoretical calculations assuming its proportionality to the electronic and nuclear-stopping power values. On the other hand, a significant reduction in the number of crystal defects was observed for irradiations performed at high temperatures or for samples annealed after irradiation. Additionally, indications of saturation of the crystal defect concentration were observed for higher fluences and the irradiation of previously defected samples.

## 1. Introduction

Silicon carbide (SiC) is suitable for various applications, including nuclear engineering, space, and electronics [1,2,3,4,5]. SiC is a hard, corrosion-resistant material that can maintain its properties even at elevated temperatures [6]. SiC decomposition occurs at a temperature of about 2545 °C [6]. As a wide-bandgap semiconductor, SiC shows promising potential in microelectronics and may serve as a viable alternative to silicon-based components under conditions far beyond the limits of Si [5]. In the nuclear industry, SiC finds application as fuel cladding, one of the layers in tristructural isotropic (TRISO) particle fuel, and as a construction material for novel very high-temperature reactors such as the Dual Fluid Reactor (DFR) [7,8]. DFR is a novel high-temperature fast reactor concept in which both the fuel and coolant are liquid and circulate in separate loops. According to one of the first designs, a metallic uranium eutectic should flow in the reactor core within large number of SiC tubes surrounded by liquid lead dealing as a coolant. The average operation temperature is assigned to be about 1000 °C. Therefore, SiC numerous applications make the material interesting to study especially at high temperatures and high neutron fluxes, which can strongly influence the mechanical stability of the structural materials.

Despite extensive research on the radiation effects in SiC dating back to the last century, there remains necessity to expand the comprehension of irradiation-induced defects even further [1]. Instead of long-lasting neutron irradiation experiments, application of heavy ion beams under well-defined conditions is much more convincing [9,10]. The advantages of using ions to emulate neutron radiations are very well-controlled conditions of irradiation, a much higher dose at the same time compared to nuclear reactors and easier access to irradiation facility [9].

During irradiation, ions lose their energy by the transfer of the kinetic energy during elastic collisions (nuclear energy loss) and by inelastic energy transfer to electrons by excitation or ionization (electronic energy loss). At higher ion energies, electron energy loss dominates, and as the energy decreases, nuclear energy loss begins to be more significant. Thus, elastic collisions occur mostly at low-energy ion bombardment and at the end of the high-energy ion path. Energy deposited in electrons can be transferred to the atoms in the lattice and influence defects dynamics. The impact of electronic energy deposition on lattice dynamics can be elucidated through three distinct mechanisms: Coulomb explosion, lattice relaxation, and the thermal spike [11,12]. Previous investigations concerning SiC indicate that the phenomenon of energy transfer between electrons and atoms in the lattice can be explained using the inelastic thermal spike model (TS) model [13,14,15]. The TS model assumes that the energy gained from electrons can be transferred to atoms via electron-phonon coupling leading to a temperature increase along the ion path. The spread of this energy depends on the electron mobility and thermal conductivity of the material [16]. Thus, metals are less sensitive to the effects of swift heavy ion irradiation as the electron energy spreads rapidly. The efficiency of the energy transfer is determined by the electron-phonon coupling, which varies for different materials. The increase in the temperature in the ion path, if sufficient, can result in melting of a surrounding region (a small cylinder or a cone shape) of the target [17]. The impact of these effects on certain materials can be the formation of ion tracks, often of the amorphous structure [17,18]. On the other hand, the energy transferred from electrons to atoms in the lattice can promote the recovery process of already distorted lattices [19].

Amorphization of SiC can be observed as an effect of the elastic collisions. SiC is insensitive for ionization and no ion track formation has been observed for electron stopping power up to 34 keV/nm [20]. Previous works have demonstrated that electronic energy loss can anneal defects in SiC during swift ion irradiation [21,22,23,24,25]. The effects of low-energy and high-energy ion irradiation are fairly well understood but the gap of knowledge occurs in intermediate energy regimes. The effect of ionization-induced annealing can be observed for Si and C ions with energies as 750 keV and 850 keV, respectively, [13]. The recent work suggests that for low $S_{e}$ values (∼1 keV/nm), the effects of thermal spike can be noticed as self-annealing behavior and recovery of existing defects [13,14,15,26].

In fast reactors, most primary recoil atoms in SiC occur at energies of several keV where nuclear-stopping power is dominant but the energies of Si or C knock-offs can reach several MeV where both nuclear and electronic stopping power may have significant effects on damage production [27]. Therefore, it is important to understand the coupled effects of nuclear and electronic energy deposition during ion irradiation.

The principal objective of this research endeavor was to pursue a comprehensive understanding of the coupled effect of nuclear and electron energy deposition from the same ion, in the same time and space. In this work, the results from experiments on 3C-SiC implanted with Si and C ions are presented with the analysis using computational methods. The energies of incident carbon and silicon ions are chosen in such a way that both nuclear and electronic stopping powers reflecting different interaction mechanisms have to be included. Irradiation was carried out at room temperature (RT) and at a high temperature of 800 °C (HT). 3C-SiC implanted samples were investigated by RBS/C. The analysis allows the determination of disorder after irradiation, and with the support of Monte Carlo simulation in the McChasy-1 code, the number of defects in the crystal lattice can be determined [28,29,30]. The experimental work is followed by the thermal spike calculation. The main point of this work is to give more understanding of the radiation effects in SiC upon the ion irradiation in the energy range, within which the energy and nuclear loss both have similar impacts on the energy transfer to the lattice.

## 2. Experimental Methods

### 2.1. SiC Samples and Irradiation Condition

3C-SiC samples were provided by NOVASIC (France) as a monocrystalline <100>-oriented layers deposited by Chemical Vapor on the Si substrate. The thickness of the SiC layer was about 10 μm. All samples were polished and cut from one wafer to 1 × 1 cm size.

Samples were irradiated at the Helmholtz-Zentrum Dresden-Rossendorf in Germany with a Tandem accelerator using ^28^Si and ^12^C ions and doses corresponding to the value of 0.01 displacements per atom (dpa) determined at a depth ∼400 nm or 0.05 dpa at a depth ∼500 nm as predicted by the SRIM code [31].

Implantations were carried out with various ion energies: 21 MeV, 5 MeV and 0.5 MeV for Si and 5 MeV, 1 MeV and 0.5 MeV for C ions, respectively. Experimental conditions are presented in Table 1. Sample 16 was separately irradiated with 0.5 MeV and 21 MeV Si beams. The maximum range of implanted ions was less than 6 μm, so the ions do not go beyond the SiC layer. The implantation was carried out at RT and HT of 800 °C and at 7° off the normal to the surface to avoid channeling effects.

### 2.2. SRIM Prediction

**Figure 1:**
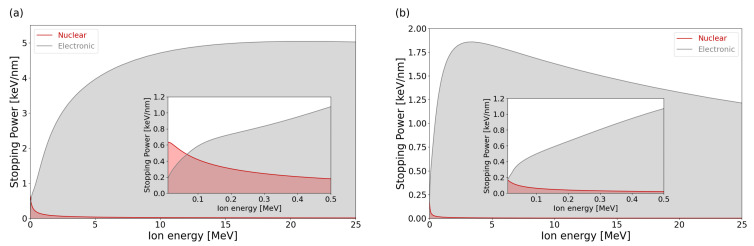
SRIM calculation showing the nuclear and electronic energy loss as a function of ion energy ((**a**) Si ion in SiC, (**b**) C ion in SiC). The region where nuclear stopping power is significant is zoomed in and shown in the inset.

**Figure 2:**
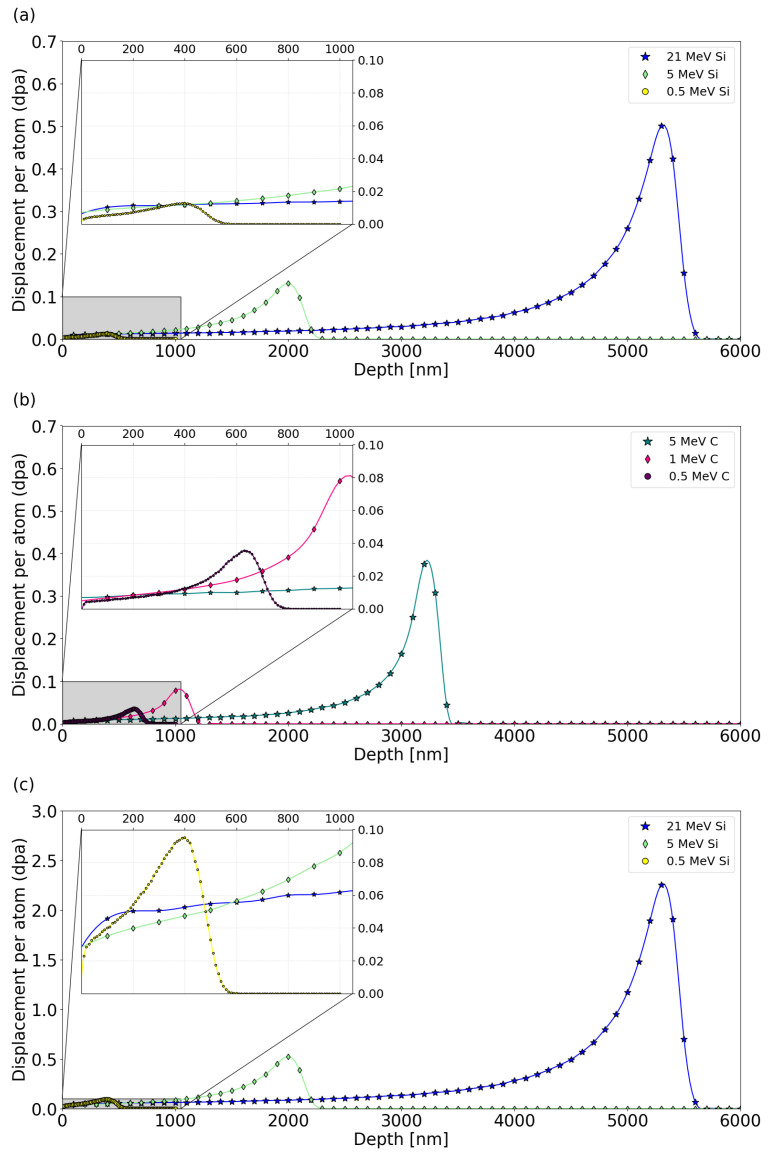
SRIM predicted damage dose (dpa) for the SiC samples irradiated with different energies of Si (**a**,**c**) and C (**b**) ions (in Table 1—samples 1–16). Inset plots represent a region investigated by RBS/C analysis (highlighted in gray).

Prediction of damage dose and energy deposition was estimated using SRIM 2008 calculation tool [31,32] with displacement energies of 35 eV and 20 eV, lattice binding energies of 3.25 eV and 2.63 eV, and surface binding energies of 4.7 eV and 7.4 eV for Si and C, respectively, [33,34]. SRIM 2008 was developed by James F. Ziegler (Annapolis, MD, USA), Jochen P. Biersack (Berlin, Germany), Matthias D. Ziegler (Los Angeles, CA, USA) and is available from www.srim.org. SiC density was set up as 3.21 g cm^−3^. Each simulation was performed in the full-cascade mode using 50,000 ions. The dpa was determined by summing up vacancies from Si and C elements from the VACANCY.txt file and replacements collisions from the NOVAC.txt file. The ion energies and fluences were chosen to represent different ratios of electronic energy deposition to nuclear energy deposition with the same damage dose. The 21 MeV Si and 5 MeV C ions reveal a high electronic energy loss, while the nuclear energy loss is negligible (see Figure 1). The choice of the irradiation fluences was made to obtain the same dose (0.01 dpa) at a depth of 400 nm (see Figure 2a,b). Additionally, the Si irradiation was performed with increased fluences to achieve the dose 0.05 dpa at depth of 500 nm (see Figure 2c).

### 2.3. RBS/C Analysis

SiC samples irradiated with Si and C ions were measured by RBS/C using a Van de Graaff accelerator with a 1.7 MeV ^4^He ion beam. The backscattered He ions were detected by a silicon surface barrier detector at an angle of 170°. A goniometer allows rotation of a sample holder in two mutually orthogonal planes (angles theta and phi). For each sample, random spectra were recorded by tilting a sample at angles θ and ϕ of −4° off the normal to the surface and consequently changing one of them within the range (−4°, +4°) with a step of 0.2°, while the other one was fixed at −4° or +4°, respectively. Such random measurements also allow a high-precision alignment of the sample along the ion beam by the indication of the main crystallographic planes. The sample orientation for the measurements in channeling mode is determined by the values of the theta and phi angles corresponding to the intersection of the crystallographic planes. RBS/C analysis allows the evaluation of disorder after irradiation. The crystalline quality of an as-grown sample was evaluated as the ratio of the backscattered yield of an aligned pristine spectrum to that of the random spectrum.

### 2.4. McChasy Code

The McChasy-1 code (Monte Carlo CHAnneling SYmulation) version 1R65 is a computational software developed by the National Center for Nuclear Research, Poland, for the interpretation of experimental data delivered by RBS/C to determine the depth distribution of damage [28,29,30]. The McChasy-1 code uses Monte Carlo simulations to reproduce trajectories of He ions in monocrystalline structures. By estimating the probability of backscattering for numerous ion–atom interactions during ongoing simulations, an RBS/C spectrum can be calculated and compared to the experimental one to determine an absolute number concentration of structural defects. To fit RBS/C spectra of irradiated samples, defect profiles should be defined prior the simulations. In this work, structural defects were modeled as randomly displaced atoms (RDAs).

## 3. Results and Discussion

### 3.1. Modeling Effects of Electronic Energy Deposition

The electronic stopping process of heavy ions in the irradiated samples can be described in two steps: ionization of atoms along the projectile path and a subsequent energy transfer from the electron gas to the lattice atoms. Within the thermal spike model, it is assumed that the electron gas can be thermalized within a short time of 10^−14^ s leading to its very high temperatures of order 10^5^ °C. This thermal energy can be then transferred to the atoms by means of electron–phonon coupling. Similar to the electron gas, the atomic subsystem can also be characterized by a thermodynamic temperature of many thousand degrees reached in 10^−13^–10^−12^ s, finally resulting in molten ion tracks. The increasing track radius can be accounted for using the diffusion equations of both electronic and atomic subsystems, as presented in Equations (1) and (2), where $T_{a}$, $T_{e}$ are lattice and electron temperature, respectively, $C_{a}$ and $C_{e}$ are lattice and electron specific heat, respectively, $K_{a}$ and $K_{e}$ are lattice and electrons heat conductivity, respectively, and *g* is the electron–phonon coupling:

(1) $$\begin{array}{c} C_{a}\left(T_{a}\right)\frac{\partial T_{a}}{\partial t}=\frac{1}{r}\frac{\partial }{\partial r}\left[rK_{a}\left(T_{a}\right)\frac{\partial T_{a}}{\partial r}\right]+g\left(T_{e}-T_{a}\right), \end{array}$$

(2) $$\begin{array}{c} C_{e}\left(T_{e}\right)\frac{\partial T_{e}}{\partial t}=\frac{1}{r}\frac{\partial }{\partial r}\left[rK_{e}\left(T_{e}\right)\frac{\partial T_{e}}{\partial r}\right]-g\left(T_{e}-T_{a}\right)+A\left(r,t\right), \end{array}$$

where *A*(*r*, *t*) is the energy deposited in the electronic subsystem for a specific energy that was calculated using the Waligorski formula for radial dose distribution and a Gaussian distribution for the time dependence (Equation (3)) [35,36,37]. $S_{e}$ is a total electronic energy loss and *b* is a normalization factor added to Equation (3) to obtain the total value of $S_{e}$ from *A*(*r*, *t*) integration:

(3) $$\begin{array}{c} A\left(r,t\right)=bS_{e}e^{\frac{-{\left(t-t_{0}\right)}^{2}}{2s^{2}}}F\left(r\right). \end{array}$$

*F(r)* is a radial distribution of the delta-electrons based on the Katz model [35]. Time $t_{0}$ is the time of the energy deposition from electrons and is equal to $10^{-15}$ s and *s* is the half-width of the Gaussian distribution, which corresponds to the time needed for electrons to reach thermal equilibrium. The electron–phonon coupling controls the energy transfer between electrons and ions and can be linked to the the mean-free path (λ) in a relationship: $g=K_{e}/\lambda^{2}$. The mean-free path is the only free parameter in Equations (1) and (2) and can be fitted to the results from experiments based on the dependence on the energy band gap [37]. For 3C-SiC, the electron mean-free path was set to 5.6 nm [25]. Electrons in SiC are considered as free electron gas with constant specific heat $\left(C_{e}\right)$ equal to 1 J cm^−3^ K^−1^ and constant electronic diffusivity $\left(D_{e}\right)$ 2 cm^2^ s ^−1^. Electron thermal conductivity is $K_{e}=C_{e}\cdot D_{e}$. In the case of electron density, the allocation of one electron per atom is taken into account. Lattice-specific heat and lattice thermal conductivity of SiC are based on Equations (4) and (5), respectively (the temperature $T_{a}$, is in Kelvin Scale) [6]:

(4) $$\begin{array}{c} C_{a}\left(T_{a}\right)=925.65+0.3772\cdot T_{a}-7.9259\cdot 10^{-5}\cdot T_{a}^{2}-3.1946\cdot 10^{7}\cdot T_{a}^{-2}, \end{array}$$

(5) $$\begin{array}{c} K_{a}\left(T_{a}\right)=\frac{1.0}{-0.0003+1.05\cdot 10^{-5}\cdot T_{a}}. \end{array}$$

In this work, TS simulations were performed using Thermal Spike GUI 2.15 code developed by C. Dufour, J. Rangama and M. Toulemonde [38,39,40]. Computations were conducted for two initial temperatures, 25 °C and 800 °C, according to the conducted experiments. The radial increment of 0.5 nm within the thermal spike was employed. The results are presented in Table 2 and Table 3. In both tables, temperatures and energies per atom are given for the center of the ion trajectory, which includes a 1 nm diameter cylinder.

**Figure 3:**
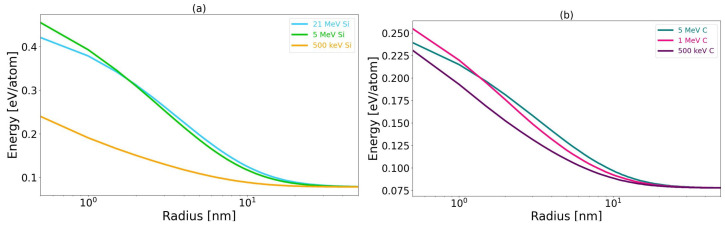
The radial distribution of energy after Si (**a**) and C (**b**) ion irradiation. Initial temperature of the SiC sample: 25 °C.

The radial distribution of the total energy per atom that is transferred to the lattice subsystem is presented in Figure 3 (results for calculation with initial temperature: 25 °C).

**Figure 4:**
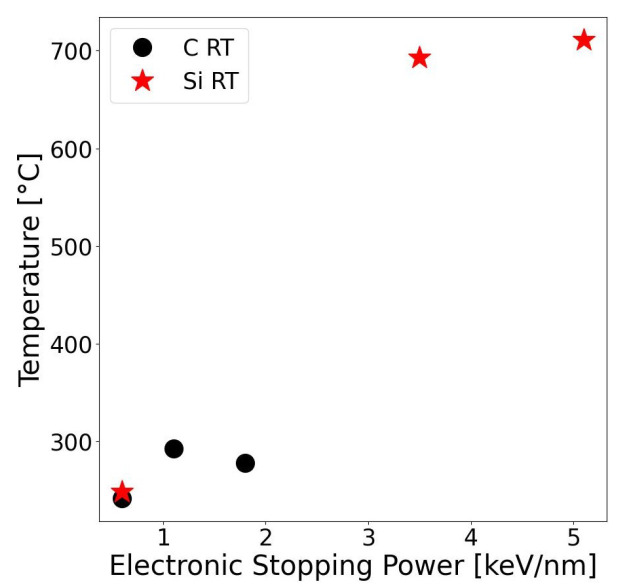
Temperature in the center of ion path as a function of electronic stopping power. Initial temperature of the SiC sample: 25 °C.

The electronic stopping power plays a crucial role in the temperature increase. The correlation of the temperature to $S_{e}$ is presented in Figure 4. It can be seen that the temperature at the central point does not increase proportionally with the increase in the $S_{e}$, but reaches the peak for certain value of $S_{e}$. Energy dissipation occurs in a larger space of the lattice for higher value of $S_{e}$.

**Figure 5:**
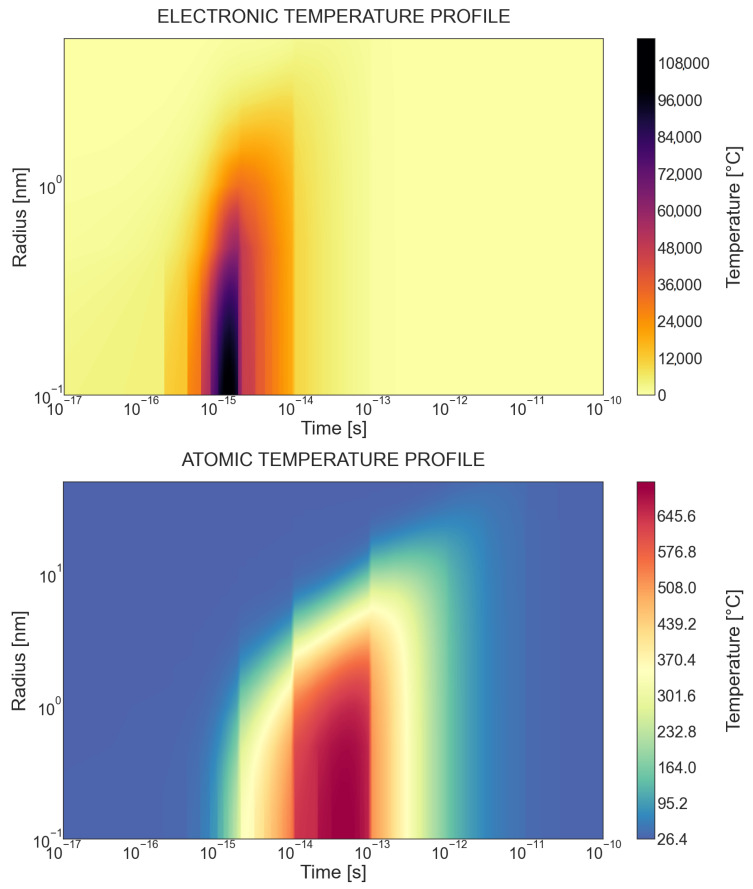
Evolution of the electronic and lattice temperatures as a function of time along the 21 MeV Si ion path. Radius is a distance from the center of the ion path. Initial temperature of the SiC sample: 25 °C.

For the case of 21 MeV Si ion irradiation, temperature profiles in electronic and atomic subsystems as a function of time are presented in Figure 5. The rapid increase in temperature in the electron system can be observed from 0.001 ps to 0.01 ps after the passage of the ion. Energy is transferred to the atomic subsystems at a time from 0.01 ps to 0.1 ps. The temperature drops to an initial value of 10 ps after the passage of the ion. The subject of interest is what effect this increase in temperature in an extremely short time and space has on the formation of defects. Molecular dynamics simulations show that in the case of irradiation with lower energy ions (where ballistic collisions dominate), the maximum peak of defects occurs after a time ∼0.1 ps. Then, the number of defects stabilizes in the time range ∼1–10 ps [41,42,43]. Thus, the peak of defects may occur after the temperature drop from the maximum value. The energy from electrons rapidly dissipates away.

### 3.2. Effect of Defects on Energy Deposition in the Center of Ion Path

The above calculation has been carried out with the assumption that electron–phonon coupling is constant and equal to 6.4·10^12^ W cm^−3^ K^−1^ (the mean free path equal 5.6 nm [25]). However the electron–phonon coupling depends on the defects in the crystal structure of SiC and can be higher by several orders of magnitude for the defective structure compared to pristine 3C-SiC [44]. For example, Smairat and Graham calculated that at RT, for 3C-SiC structure with a vacancy concentration of 12.5% electron–phonon coupling can be equal 10^14^ W cm^−3^ K^−1^ and increases as the electron gas temperature increases [44].

**Figure 6:**
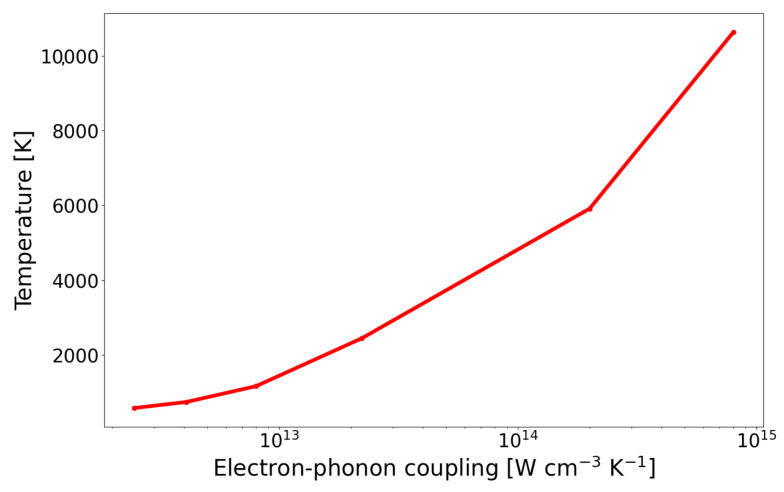
The maximum temperature in the center of the ion path as a function of electron–phonon coupling.

The dependence of the maximum temperature of the ion path as a function of the electron–phonon coupling is shown in Figure 6. Taking into account changes in the electron–phonon coupling with the number of defects in the calculations shows how strongly the defects may affect the temperature along the ion path. In a damaged structure, the energy transferred to the lattice may be enough for a phase change, and later, recrystallization is possible. The explanation of the repair mechanism is still an open question.

### 3.3. RBS/C Analysis

The crystalline quality of the as-grown sample was first evaluated by calculating the minimum yield $\chi_{min}$, which is the ratio of the backscattering yield for the aligned spectrum to the random one, being less than 3%. This denotes the high crystal quality of the samples. Energies of backscattered He-ions depend on the mass of target atoms and on the angle of detection. The maximum energies corresponding to the backscattering from the surface at 170° are 961.7 keV for Si and 429.5 keV for C atoms, respectively. The detected energy of the He-ions backscattered at a certain depth in the crystal decreases due to the energy loss occurring on the way in and out.

**Figure 7:**
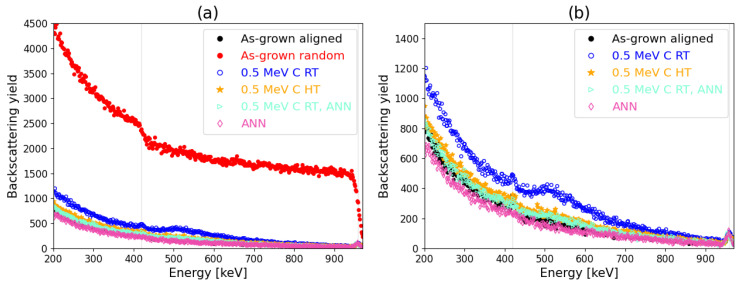
RBS/C spectra for different 500 keV C irradiation conditions. The reference random spectrum is shown in (**a**), and it is omitted in (**b**) for the sake of clarity while the plot area in (**b**) is also zoomed in. Blue dots denote irradiation at RT, whereas orange stars represent irradiation at HT. HT annealing (8 min) after RT irradiation is represented by aquamarine triangles and HT annealing (8 min) is represented by pink diamonds. RBS/C spectra for different 500 keV C irradiation conditions. The reference random spectrum is shown in (**a**), and it is omitted in (**b**) for the sake of clarity, while the plot area in (**b**) is also zoomed in.

**Figure 8:**
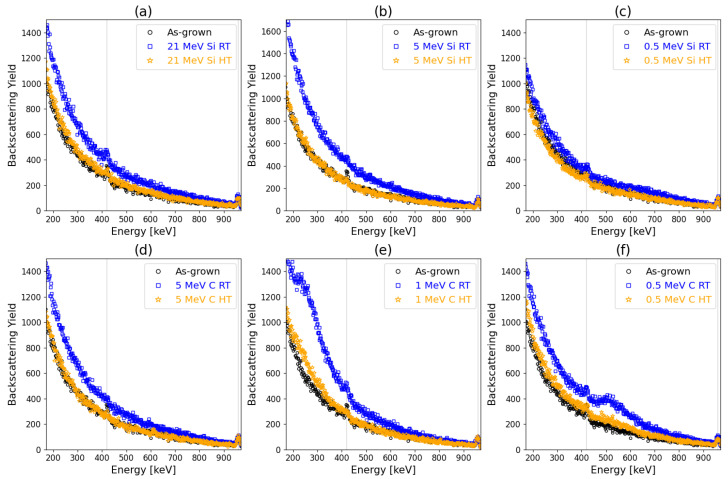
RBS/C spectra for the samples irradiated at room temperature (RT) and high temperatures (HT) using (**a**) 21 MeV Si, (**b**) 5 MeV Si, (**c**) 0.5 MeV Si, (**d**) 5 MeV C, (**e**) 1 MeV C, and (**f**) 0.5 MeV C ions, respectively.

**Figure 9:**
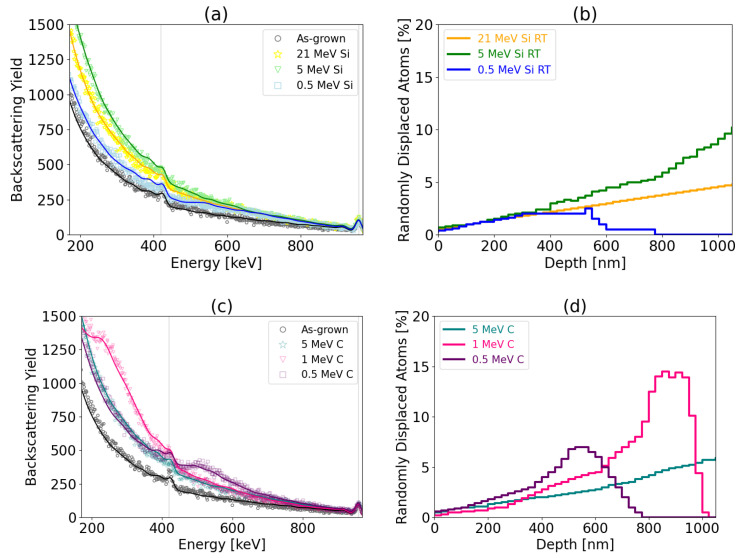
The results for irradiation at RT using Si (**a**,**b**) and C (**c**,**d**) ions (0.01 dpa at the depth of 400 nm). On the left, RBS/C spectra (discrete plots) and spectra simulated by McChasy code (solid lines) are shown. The corresponding randomly displaced atoms determined by the McChasy simulations are on the right.

**Figure 10:**
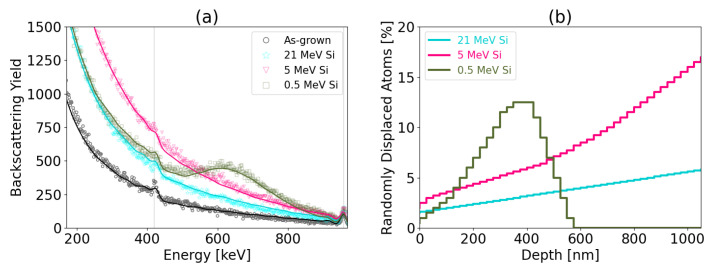
Results of irradiation performed with Si ions (0.05 dpa at the depth of 500 nm). (**a**), RBS/C spectra (discrete plots) and spectra simulated by McChasy code (solid lines) are shown. The corresponding randomly displaced atoms determined by the McChasy simulations are (**b**).

**Figure 11:**
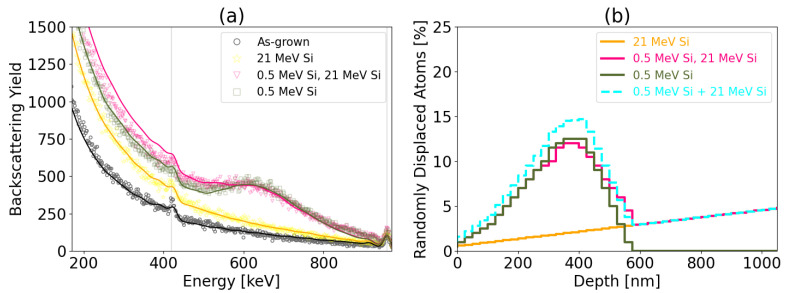
RBS/C spectra as discrete plots and channeling spectra fitted by McChasy code as solid lines (**a**) and corresponding randomly displaced atoms (**b**) for Si irradiation. The turquoise dotted stepline corresponds to the sum of RDA produced by 0.5 MeV Si and 21 MeV Si, separately.

Aligned spectra corresponding to the as-grown (pristine) and irradiated samples as well as a reference random spectrum are shown in Figure 7, Figure 8, Figure 9, Figure 10 and Figure 11 referring to the implantation using C and Si ions. Figure 7 shows the spectra recorded for the pristine and as-implanted samples and the results of the thermal annealing applied after or during the corresponding irradiation. The random spectrum is shown only in Figure 7a, while it was omitted for the sake of clarity in Figure 7b as well as Figure 8, Figure 9, Figure 10 and Figure 11. Figure 8 shows the effects of irradiation temperature on the formation of defects. Figure 8a–c show the results for Si irradiation, while Figure 8d–f present results for C irradiation.

Thermally induced recovery was studied by heating the as-grown sample and those irradiated by 0.5 MeV C ions to 800 °C (Figure 7). The annealing time was the same as the irradiation time. Heating after irradiation reduces defects almost the same as annealing during irradiation. It can be observed that the sample with 0.5 MeV C ion irradiation at RT followed by heating at 800 °C has a similar RBS/C spectrum as the sample with 0.5 MeV C ion irradiation at 800 °C. Dynamic annealing (during irradiation) led to the annealing of lattice disorder similar to the annealing after irradiation. After irradiation at HT, no peaks due to C surface were observed. The heating of the as-grown sample suggests a small number of existing defects.

RBS/C spectra of the Si and C-irradiated samples as a function of irradiation temperature and ion energy are shown in Figure 8. The backscattering yield changes with the irradiation temperature. At RT, damage is noticeable in each case, while at HT, measured damages are reduced. Following high-temperature irradiation of Si ions, the absence of discernible defects is observed.

RBS/C spectra recorded for the 3C-SiC samples before and after irradiation using Si and C ions are shown in Figure 9a,c, respectively. Corresponding depth distributions of RDAs calculated by the McChasy-1 code are shown in Figure 9b,d. The damage profiles show evidence of existing defects that are in good agreement with the dpa profiles predicted by SRIM. The backscattering yield of the aligned spectra increase with the fluence of Si ions (Figure 9a). In the case of C irradiation, damage peaks are formed for energies of 0.5 MeV and 1 MeV, corresponding to the depths 550 nm and 900 nm. They are a consequence of stopping C ions at very low energies for which the nuclear stopping power is significant.

The effects of irradiation temperature on defect production can be clearly seen when comparing RT to HT irradiation. The irradiation temperature was chosen to have a similar value as a temperature that can be reached in the ion path regarding thermal spike prediction. Here, no effects of temperature increase due to electronic stopping power can be noticed during low-dose irradiation (0.01 dpa). The defects production is linear from the surface to the measured depth of 1050 nm for 21 MeV Si and 5 MeV C, where the electron stopping power is dominant, and the peak of damage is out of the RBS/C analysis range. Although previous research suggested that full suppression may occur to some depth, this research does not notice that for 21 MeV Si or 5 MeV C [14,15]. The increase in temperature according to the thermal spike model occurs in an extremely short timescale and limited radius. Thus, the recovery is limited to pre-existing damage.

RBS/C spectra for Si irradiation with dose of 0.05 dpa are presented in Figure 10. At a depth of 500 nm where the same dose is reached for various ion energies, irradiation of 21 MeV Si causes fewer defects than in the case of 5 MeV Si and 0.5 MeV Si, which shows that defect saturation occurs. The saturation of defect formation after receiving a certain dose is a result of the fact that annealing of defects occurs before damage cascades are created. Table 4 shows the RDA number predicted for different ion energies at 0.01 dpa and 0.05 dpa, respectively. At dose 0.01 dpa, the RDA values are similar for all ions energies, while in case of irradiation at 0.05 dpa, the RDA number is two times smaller for 21 MeV Si than for 5 MeV Si or 0.5 MeV Si.

Pre-damage samples with 0.5 MeV Si ions were additionally irradiated with 21 MeV Si ions (Figure 11). The total damage number in pre-damage sample does not increase after irradiation with 21 MeV and the reduction of defects in the damage peak occur. This confirms that pre-existing defects can increase effects of damage annealing or recovery.

## 4. Conclusions

This paper investigates the coupled effects of nuclear and electronic energy deposition during ion irradiation. Developing a physical understanding of ion radiation damage in SiC is important for using ions to simulate neutron radiation damage.

This research focused on temperature effects on Si and C radiation damage in 3C-SiC. Irradiation carried out at HT causes annealing of defects, which was demonstrated in the performed experiment. During ion irradiation at RT, annealing of defects can also occur due to local heating of the samples after transferring deposited electronic energy to lattice atoms due to electron–phonon coupling. The electron-stopping power and nuclear-stopping power affect defect dynamics independently, due to a little shift in the timescale. The results show no effect of the temperature increase caused by the thermal spike on defect production during low-dose irradiation, while with an increasing dose, the defect suppression can be observed for the higher energy irradiation (21 MeV). The explanation for this is the occurrence of annealing of pre-existing defects. With higher fluency, the probability of ion passage close to the damaged area increases and the ion paths overlap.

In this work, the calculations based on the thermal spike model of temperature along the ion path during ion passage were performed. Calculations show a high-temperature dependency in the ion path on the defects number in 3C-SiC. The results suggest that as the defect number increases, the temperature in the ion path rises, and thus, the possibility of repairing the crystal lattice becomes greater. This is confirmed by experimental results, where no additional defects were measured after 21 MeV Si irradiation (0.01 dpa) on the pre-damage sample, while in the pristine sample, defects can be detected after irradiation with 21 MeV Si with the same dose (0.01 dpa).

The results show that annealing and repair effects are important for the prediction of radiation damage in SiC. Thus, the understanding of the mechanism of this phenomena is important for modeling the neutron radiation in SiC. On the other hand, the repair mechanism occurring in SiC supports the use of this material in high-temperature reactors such as the Dual Fluid Reactor.

## Figures and Tables

**Table 1:** Experimental condition.

Sample	Ion	Energy [MeV]	Fluence [$10^{13}$cm^−2^]	Flux [$10^{11}$s^−1^cm^−2^]	Temperature [°C]
1	Si	21.0	100	1.60	25
2	Si	21.0	100	2.30	800
3	Si	5.0	25	0.63	25
4	Si	5.0	25	3.90	800
5	Si	0.5	2	0.50	25
6	Si	0.5	2	0.94	800
7	C	5.0	200	2.30	25
8	C	5.0	200	2.30	800
9	C	1.0	40	5.90	25
10	C	1.0	40	5.90	800
11	C	0.5	15	3.10	25
12	C	0.5	15	3.10	800
13	Si	21.0	450	1.60	25
14	Si	5.0	100	2.50	25
15	Si	0.5	15	3.30	25
16 ^I^	Si	0.5	15	3.10	25
16 ^II^	Si	21.0	100	1.60	25

Two irradiations were performed on sample 16, first irradiation with 0.5 MeV (16 ^I^), and subsequent irradiation with 21 MeV (16 ^II^).

**Table 2:** Temperatures and energies in the center of the ion trajectory calculated from inelastic thermal spike model. Initial temperature of the SiC sample: 25 °C.

Ion	Ion Energy [MeV]	Electronic Stopping Power [keV/Å]	Temperature [°C]	Energy per Atom [eV]
Si	21.0	0.51	711	0.22
Si	5.0	0.39	693	0.22
Si	0.5	0.11	249	0.12
C	5.0	0.18	278	0.12
C	1.0	0.15	293	0.13
C	0.5	0.11	242	0.12

**Table 3:** Temperatures and energies in the center of the ion trajectory calculated from inelastic thermal spike model. Initial temperature of the SiC sample: 800 °C.

Ion	Ion Energy [MeV]	Electronic Stopping Power [keV/Å]	Temperature [°C]	Energy per Atom [eV]
Si	21.0	0.51	1554	0.48
Si	5.0	0.39	1554	0.48
Si	0.5	0.11	1076	0.35
C	5.0	0.18	1103	0.36
C	1.0	0.15	1124	0.36
C	0.5	0.11	1066	0.35

**Table 4:** Randomly displaced atoms for Si irradiation.

Ion	Ion Energy [MeV]	Electronic Stopping Power [keV/Å]	Damage Dose [dpa]	Randomly Displaced Atoms [%]
Si	21.0	0.51	0.01	2.2
Si	5.0	0.35	0.01	2.4
Si	0.5	0.80	0.01	2.0
Si	21.0	0.51	0.05	3.5
Si	5.0	0.35	0.05	6.8
Si	0.5	0.80	0.05	7.0

## Data Availability

Data are contained within the article.

## Abbreviations and Symbols

SiC	Silicon Carbide,
TRISO	Tristructural Isotropic Particle Fuel,
DFR	Dual Fluid Reactor,
RBS/C	Channeling Rutherford Backscattering Spectrometry,
TS	thermal spike,
RT	room temperature,
HT	high temperature,
$S_{e}$	electronic energy loss,
dpa	displacements per atom,
McChasy-1	Monte Carlo CHAnneling SYmulation code,
RDAs	Randomly Displaced Atoms,
$T_{a}$	lattice temperature,
$T_{e}$	electron temperature,
$C_{a}$	lattice specific heat,
$C_{e}$	electron specific heat,
$K_{a}$	lattice heat conductivity,
$K_{e}$	electrons heat conductivity,
*g*	electron-phonon coupling
*A*(*r*, *t*)	the energy deposited in the electronic subsystem,
*b*	a normalization factor,
*F*(*r*)	a radial distribution of the delta-electrons,
$t_{0}$	the time of the energy deposition from electrons,
*s*	the half-width of the Gaussian distribution,
λ	the mean-free path,
D_e	electronic diffusivity,
$\chi_{min}$	minimum yield.
